# Potato Tuber Chemical Properties in Storage as Affected by Cultivar and Nitrogen Rate: Implications for Acrylamide Formation

**DOI:** 10.3390/foods9030352

**Published:** 2020-03-18

**Authors:** Na Sun, Yi Wang, Sanjay K. Gupta, Carl J. Rosen

**Affiliations:** 1 Department of Soil, Water, and Climate, University of Minnesota, St. Paul, MN 55108, USA; sunxx891@umn.edu (N.S.); gupta020@umn.edu (S.K.G.); 2 Department of Horticulture, University of Wisconsin, Madison, WI 53706, USA; wang52@wisc.edu

**Keywords:** reducing sugars, glucose, asparagine, acrylamide, potato, cultivar, storage time

## Abstract

Recently released potato cultivars Dakota Russet and Easton were bred for low reducing sugars, and low acrylamide-forming potential in French fries. The objectives of this study were to determine: (1) the effects of nitrogen rate and storage time on tuber glucose concentrations in different cultivars; (2) the relationships between acrylamide, glucose, and asparagine for the new cultivars and Russet Burbank. The study was conducted at Becker, Minnesota over a period of two years on a loamy sand soil under irrigated conditions. All cultivars were subjected to five N rates from 135 to 404 kg ha^−1^ in a randomized complete block design. Following harvest, tubers were stored at 7.8 °C and sampled at 0, 16, and 32 weeks. Dakota Russet and Easton had significantly lower concentrations of stem- and bud-end glucose, asparagine, and acrylamide than those of Russet Burbank in both years. The effect of storage time on glucose concentration was significant but differed with cultivar and year. N rate effects on stem- and bud-end glucose concentrations were cultivar and storage time dependent. After 16 weeks of storage, both asparagine and acrylamide concentrations linearly increased with increasing N rate. Glucose concentration was positively correlated with acrylamide concentration (*r*^2^ = 0.61). Asparagine concentration was also positively correlated with acrylamide concentration (*r*^2^ = 0.45) when the asparagine:glucose ratio was <1.306. The correlation between fry color and stem-end glucose concentration was significant over three cultivars in both years, but stronger in a growing season with minimal environmental stress. Taken together, these results suggest that while acrylamide formation during potato processing is a complex process affected by agronomic practices, environmental conditions during the growing season, and storage conditions, cultivar selection may be the most reliable method to minimize acrylamide in fried products.

## 1. Introduction

Acrylamide, a neurotoxin and probable carcinogen for humans, was first reported in fried potato (*Solanum tuberosum* L.) products in 2002 [1]. The concentration of acrylamide in processed products is strongly affected by processing conditions (for instance frying temperature and duration) and the concentrations of acrylamide precursors, reducing sugars, and asparagine [2,3]. Other factors such as cultivar, soil nutrition, environmental conditions during plant growth, harvesting time, storage conditions and genetic modification can affect the concentrations of reducing sugars and asparagine, and consequently acrylamide-forming potential [4,5,6,7,8,9,10,11,12,13]. Following the evaluation of numerous approaches to mitigate acrylamide in fried potato products, progress has been made in lowering its concentration. Power et al. [14] reported the mean acrylamide level decreased from 763 µg kg^−1^ in 2002 to 358 µg kg^−1^ in potato chips from 20 European countries; Wang et al. [15] showed that many new elite U.S. fry processing cultivars exist with substantially lower acrylamide-forming potential than the standard check Russet Burbank. However, despite the current mitigation efforts, acrylamide remains a public concern due to the potential cancer risk [16].

Nitrogen (N) management is a common agronomic practice that can influence acrylamide precursors, reducing sugars, glucose and fructose, and asparagine in potato tubers, and consequently acrylamide-forming potential. The effects of N rate on reducing sugars in potato tubers can be highly variable. For example, reducing sugar concentrations have been reported to both increase or decrease with increasing N supply in one study, while in another study N rate had no effect on reducing sugars [5,10]. A few studies also reported a decrease in reducing sugars at the stem end or in the whole tuber with increasing N rate [6,17,18,19]. Compared to the complex responses of reducing sugars to N rate, asparagine concentrations have been shown to generally increase with increasing N rate [19,20,21]. Low tuber reducing sugars concentrations were detected along with high asparagine concentrations in these studies [20,21].

Tuber glucose concentrations can vary significantly during storage and often interact with cultivar and growing conditions [22,23,24,25]. Muttucumaru et al. [24] reported reducing sugars’ concentrations increased from 2 to 6 months of storage at 8 °C for tubers harvested from one location but decreased for tubers harvested from the other location under the same storage conditions for the cultivars Pentland Dell and Umatilla Russet. In another study, tuber glucose concentrations increased during the 9-month storage in one year but were not affected by storage time in the following year at a storage temperature of 7.2 °C for Alpine Russet [25]. The results of these studies suggest that environmental conditions during the growing season can alter or even reverse the storage time effect on reducing sugars under appropriate storage temperature conditions for certain cultivars.

Unlike the variable responses of reducing sugars to storage time, asparagine concentration is generally considered stable during storage [26,27,28]. Olsson et al. [29] investigated the asparagine content fluctuation during the long-term storage at both 3 and 10 °C for eight potato cultivars. Minimal effects of storage time and storage temperature on asparagine concentration were reported in the study and genetic and year effects were substantial for some cultivars. Matsuura-Endo et al. [30] reported minor variation of tuber asparagine concentrations during 18 weeks of storage at 2–18 °C.

The relationship between reducing sugars, asparagine, and acrylamide is complicated and appears to be affected by the relative concentrations of the precursors [31]. Reducing sugar concentrations are generally considered the limiting factor for acrylamide formation due to their lower concentration than asparagine in fry-processing potato tubers [26]. This relationship often results in a significantly positive correlation between reducing sugar and acrylamide concentrations [15,23,27,32,33]. However, positive correlations between asparagine and acrylamide concentrations have also been reported [30,34]. Shepherd et al. [35] suggested that both reducing sugars and asparagine should be considered to help explain the variation in acrylamide concentrations. Muttucumaru et al. [36] speculated that higher amounts of reducing sugars relative to asparagine in some cultivars were the reason for a more significant role of asparagine in acrylamide formation. They found that asparagine affected acrylamide formation when its concentration was 2.257× lower than that of reducing sugars in 20 potato cultivars grown in two locations after 2 and 6 months of storage [24].

This study investigates the effects of N fertilization rate on asparagine and glucose concentrations in recently released potato cultivars during storage and implications for acrylamide formation during processing over two growing seasons. The specific objectives of this study were to: (1) determine the effects of N rate and storage time on stem- and bud-end glucose concentrations of Easton and Dakota Russet cultivars, relative to the standard cultivar Russet Burbank over two growing seasons; and (2) elucidate relationships between acrylamide, glucose, and asparagine, for the test cultivars after 16 weeks of storage at 7.8 °C.

## 2. Materials and Methods

The study was conducted at the Sand Plain Research Farm in Becker, Minnesota on a Hubbard loamy sand soil (sandy, mixed, frigid Entic Hapludolls) in 2014 and 2015. A randomized complete block experimental design was adopted with a factorial treatment arrangement of N rate and cultivar replicated four times. Three French fry cultivars—Russet Burbank, Dakota Russet, and Easton—were subjected to five N fertilizer treatments, 135, 202, 269, 336, and 404 kg ha^−1^. For each treatment, 101 kg ha^−1^ N fertilizer was applied pre-planting as polymer coated urea (44–0–0; Environmentally Smart Nitrogen, Agrium, Inc.) and 34 kg ha^−1^ N fertilizer was applied at planting as diammonium phosphate (18–46–0). The remainder, 0, 67, 134, 201, and 269 kg N ha^−1^, for each treatment was applied at emergence as polymer coated urea.

Each plot consisted of seven 7.6 m rows with 25 plants in each row. The spacing between rows was 0.9 m and seed tubers were spaced 0.3 m apart within each row. Sample tubers were harvested from rows 4 and 5. Whole “B” seed (56–84 g) of Russet Burbank and cut “A” seed (56–84 g) of Dakota Russet and Easton were hand planted in furrows on 6 May, 2014 and 21 April, 2015. Soil properties and further cultural practices used in this study can be found in [37].

### 2.1. Sample Collection and Analysis

Tuber harvest dates were scheduled on 2 October 2014 and 28 September 2015 according to weather conditions. After harvest, approximately 23 kg of tubers (single tuber weight between 170 and 283 g) from each plot were shipped to the USDA-ARS (United States Department of Agriculture-Agricultural Research Service) Potato Research Worksite in East Grand Forks, Minnesota. Tubers were preconditioned at 10 °C for two weeks and then stored at 7.8 °C for 32 weeks. Glucose concentrations in both the stem and bud end of tubers were determined by a YSI-2700 Select Biochemistry Analyzer (Yellow Springs Instrument Co. Inc. Yellow Springs, Ohio, USA) after 0, 16, and 32 weeks of storage. Glucose extraction from tubers was as follow: stem-end samples (50 g per sample) were collected from the 3.8 cm of tuber tissues surrounding the stem scar, while bud-end samples (50 g per sample) were collected from the remainder of the tuber. The stem- and bud-end samples were ground separately in an Acme Juicerator (Acme Equipment, Spring Hill, FL, USA), and brought up to a final volume of 100 mL with 50 mM phosphate buffer (pH 7.2) in a beaker. Samples were then stored at 4 °C for 20–30 min and gently stirred without disturbing the precipitate (including starch) in the bottom of the beaker. For each sample, 15 mL of juice was transferred into a labeled scintillation vial and frozen for glucose analysis.

Tuber samples for asparagine analysis were collected after vine kill both years. Six tubers greater than 85 g from each plot were randomly chosen for the determination of asparagine concentration. Fresh tuber tissue was collected about 0.5 cm from the stem and bud ends of the tubers using a 7.8 mm Humboldt Brass Cork borer, and then stored at −20 °C for later asparagine extraction. Asparagine was determined by liquid chromatography with tandem mass spectrometry. Details on sample preparation and determination of asparagine analysis in this study can be found in Sun et al. [37]. All mass spectrometry analyses were conducted by the Center for Mass Spectrometry and Proteomics at the University of Minnesota.

To investigate the relationships between acrylamide formation and its precursors, we selected the storage midpoint of 16 weeks, with the assumption that asparagine concentrations analyzed at harvest were similar to those over the entire storage period [29,30]. After 16 weeks of storage, tubers were fried at the worksite in East Grand Forks. Five tubers from each plot were washed, cut (cross section dimension: 22 × 6.5 mm), blanched at 74 °C for 6 min, and then par fried in canola oil at 185 °C for 2.75 min. Even though a frying temperature of 168 °C has been recommended to lower acrylamide formation in processed potato products, our objective was to access the contribution of the precursors, asparagine and glucose, on acrylamide formation in new potato cultivars under a commercial setting. Therefore, we adopted a French fry production procedure currently used by the industry. In commercial French fry production, blanching is performed to remove precursors in the uncooked cut potatoes, which will then reduce acrylamide formation during the frying process. After freezing at −26 °C, all fries were finish fried for 1 min at 191 °C. Fry color at stem and bud ends was determined at the East Grand Forks worksite with a Photovolt Reflectance Meter (Photovolt Instruments Inc., Minneapolis, MN, USA) about 3 min after finish frying. The fried samples were shipped frozen to the University of Minnesota for acrylamide extraction. For each plot, three fries were ground for 30 s in a coffee grinder, and 0.8–1.0 g of ground powder was taken for each sample for acrylamide extraction and determination as described by Sun et al. [25]. Acrylamide was determined in whole fries and, therefore, the average concentrations of stem- and bud-end glucose and asparagine were used to explore the relationships between these precursors and acrylamide formation.

### 2.2. Statistical Analysis

Analysis of variance (ANOVA) for glucose in the stem and bud end as a function of N rate, cultivar, storage time, and year was conducted using PROC ANOVA (Analysis of Variance Procedure) with repeated measures for storage time in SAS 9.4 statistical software package (Statistical Analysis System, SAS Institute Inc., Cary, NC, USA). A square root transformation was used when necessary to account for the heterogeneity of variance. The average of stem-end and bud-end glucose or asparagine concentrations was considered as the concentration of the whole-tuber glucose or asparagine, which was then analyzed with PROC ANOVA as a function of N rate, cultivar, and year. Acrylamide concentrations in fried potatoes were also analyzed using PROC ANOVA. Means of interest were compared using the least significant difference (LSD) test at the 5% probability level. PROC GLM (General Linear Models Procedure) and CONTRAST statements were used to determine linear or quadratic effects of N rate on glucose, asparagine, and acrylamide concentrations. All figures and tables were depicted using Excel (Microsoft, Seattle, WA). A *p*-value < 0.05 was considered significant. The relationship between acrylamide and the molar ratio of asparagine to glucose was investigated using a piecewise linear regression (also known as a broken-stick regression) model:

acrylamide = a_1_ + b_1_ × asparagine/glucose for asparagine/glucose ≤ c (1)

acrylamide = a_2_ + b_2_ × asparagine/glucose for asparagine/glucose > c (2)

where a_1_ and a_2_ are the intercept, b_1_ and b_2_ are the slope of the two linear lines, and c is the breakpoint in the model [24,38]. PROC NLIN (Nonlinear Regression Models Procedure) statement was used to estimate the parameters a_1_, a_2_, b_1_, b_2_, and c in SAS.

## 3. Results and Discussion

### 3.1. Glucose Concentrations

**Figure 1:**
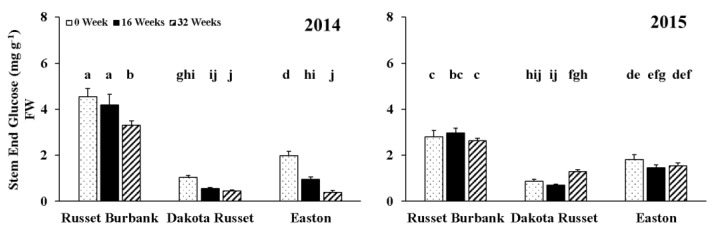
Three-way interaction of cultivar by storage time by year effect on stem-end glucose concentrations in 2014 and 2015 (Means were separated at the 5% level, with the same letter above the bar indicating no significant difference.). FW: Fresh weight.

Stem-end glucose concentrations in 2014 and 2015 were significantly influenced by the interaction of cultivar by storage time by year (Table 1). A decrease in glucose concentration at the stem end was observed for all three cultivars during the 32-week storage in 2014 (Figure 1). The decrease of stem-end glucose concentration was significant at harvest for Easton, and 16 weeks after storage for Russet Burbank and Dakota Russet in 2014. In 2015, the fluctuation of stem-end glucose during the 32-week storage varied by cultivar (Figure 1). For Dakota Russet, the stem-end glucose concentration was stable for the first 16 weeks and significantly increased since then. However, Russet Burbank and Easton had stem-end glucose concentrations not affected by storage time in 2015. Stem-end glucose concentrations of Dakota Russet and Easton were significantly lower than that of Russet Burbank during the 32-week storage both years.

**Figure 2:**
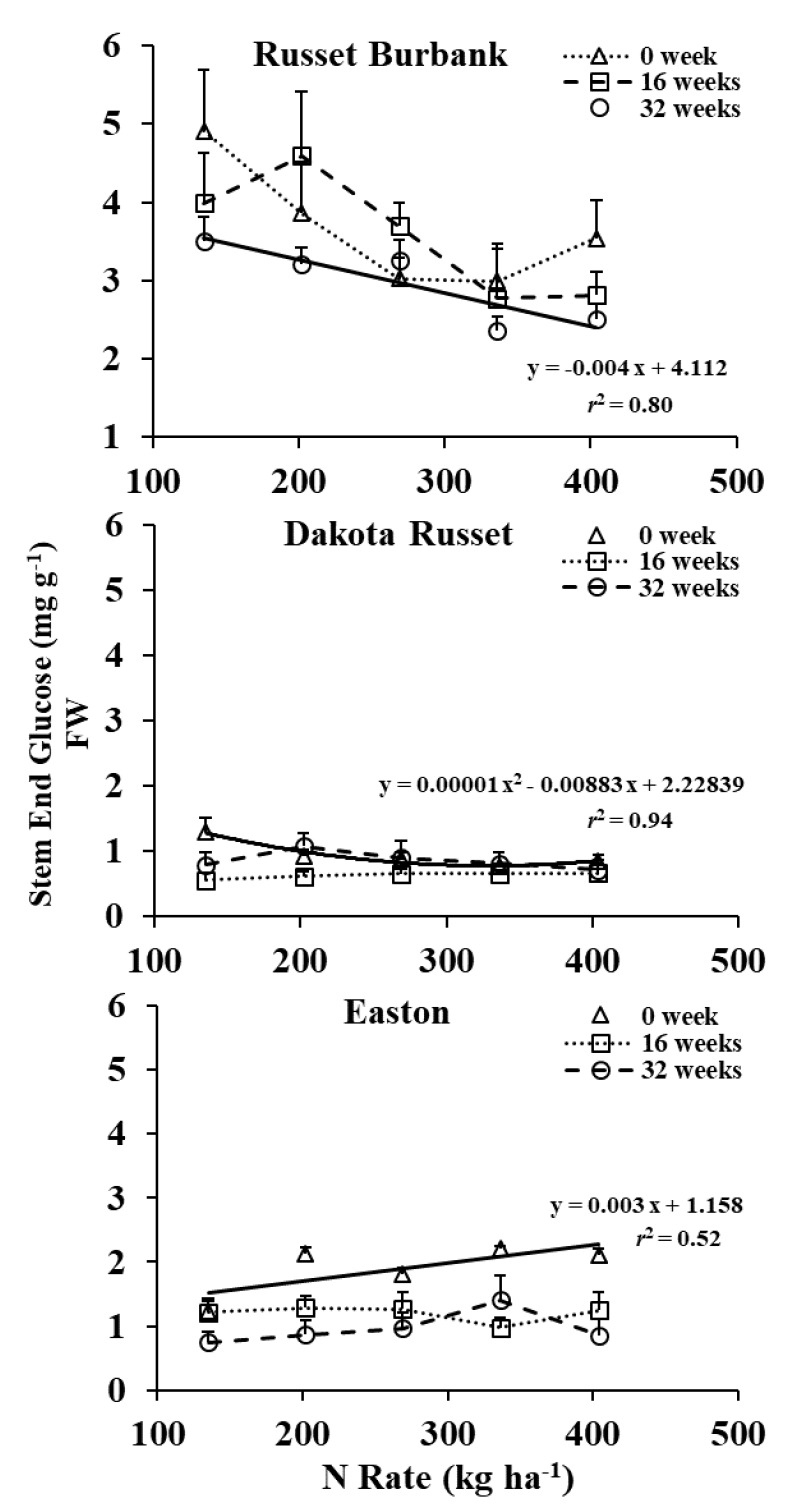
Three-way interaction of cultivar by N rate by storage time on stem-end glucose in 2014 and 2015.

The effect of N rate on stem-end glucose concentration was storage-time and cultivar dependent (Table 1 and Figure 2). Stem-end glucose concentration in Russet Burbank tended to decrease with increasing N supply through the entire storage. However, the N rate effect was significant at 32 weeks of storage only. Stem-end glucose concentrations of Easton and Dakota Russet responded to N supply at harvest only, quadratically decreased for Dakota Russet and linearly increased for Easton. Overall, the N rate effect on stem-end glucose concentration of Easton and Dakota Russet was not as dramatic as it was for Russet Burbank.

**Figure 3:**
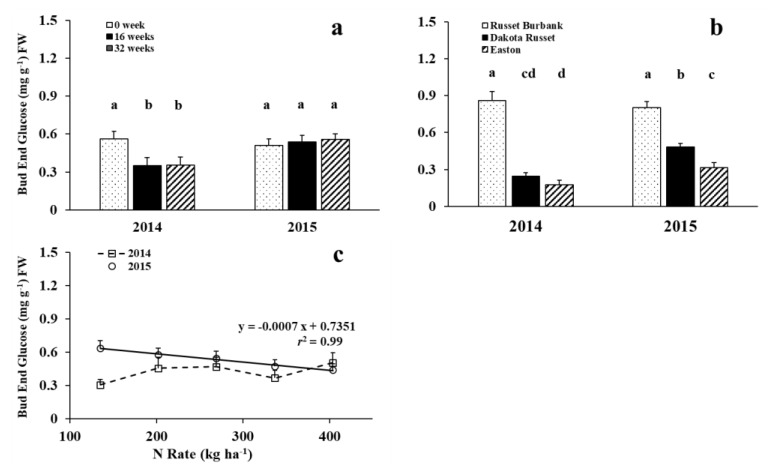
Interactions of storage by year (**a**), cultivar by year (**b**), and N rate by year (**c**) effects on bud-end glucose (Means were separated at the 5% level, with the same letter above the bar indicating no significant difference.).

Bud-end glucose concentration of all three cultivars was significantly affected by storage time, cultivar, and N rate, but the effect differed by year (Table 1 and Figure 3). Bud-end glucose concentration averaged over cultivar and N rate decreased and then leveled off at 16 weeks of storage in 2014, but it was not affected by storage time in 2015 (Figure 3a). New cultivars Dakota Russet and Easton had significantly lower bud-end glucose concentrations than Russet Burbank in both years. However, bud-end glucose concentrations of the new cultivars significantly increased in 2015, and the differences between these two cultivars were greater with Dakota Russet than Easton in 2015 (Figure 3b). Bud-end glucose concentrations were not affected by N supply in 2014, but linearly decreased with increasing N rate in 2015 for all three cultivars averaged during the entire storage (Figure 3c). The results indicate that the effect of environmental conditions during the growing season on bud-end glucose concentration continues in storage and can be affected by N rate and cultivar.

Cold temperatures before vine kill may have induced reducing sugar accumulation in both stem and bud end for all three cultivars at harvest in 2014, while no cold stress occurred in 2015 [37]. Even though tubers were preconditioned at 10 °C for two weeks after harvest, higher glucose concentrations were still detected at the stem end for Russet Burbank in 2014 (4.54 mg g^−1^) than in 2015 (2.79 mg g^−1^), suggesting that two weeks was not long enough for preconditioning of this cultivar. Dakota Russet and Easton also had elevated stem-end glucose at harvest in 2014 relative to 2015 (20% higher for Dakota Russet and 9% higher for Easton), but the difference was not as large as in Russet Burbank (63%), suggesting a stress resistant characteristic of the new cultivars.

Glucose concentrations decreased in the stem and bud end of all three cultivars during storage in 2014, which may be due to the proper storage temperature (7.8 °C) for the tuber reconditioning in this study. This is consistent with the conclusion of Silva and Simon [39], who reported a decrease of glucose concentration from 2.64% to 0.58% dry weight (DW) (averaged over seven cultivars) after tuber reconditioning at 15 °C for 2 weeks during storage.

Knowles et al. [22] stored Ranger Russet, Umatilla Russet, and Russet Burbank at low temperatures (4.5 and 6.7 °C) and reported an increase in reducing sugar accumulation during the first 31 days. However, a decrease in reducing sugar concentrations was observed over the next 220 days when the storage temperature increased from 4.5 to 6.7 °C and 6.7 to 9 °C for all three cultivars. The results above suggest that sugar accumulation from cold stress is reversible with proper reconditioning temperatures.

The effect of N rate on glucose accumulation differed in stem and bud end, and often interacted with cultivar, storage time, or year. Contradictory results of N rate effects on glucose concentrations have been reported in the previous studies [10,17,40,41,42,43]. Westermann et al. [17] reported an increase in reducing sugars at the bud end, and a decrease at the stem end with increasing N supply from 0 to 336 kg ha^−1^ for Russet Burbank, which is consistent with the results for the stem end in this study. However, a minimal N rate effect on reducing sugar concentration for Russet Burbank was reported by Zebarth et al. [41]. Knowles et al. [43] reported a decreasing tendency of reducing sugars with increasing N supply at the stem and bud end for Alpine Russet, while reducing sugars of the cultivar Sage Russet barely responded to N supply at harvest. Gause [44] reported reducing sugar concentrations in Easton were not significantly affected by N rate when stored at 10 °C, which agreed in part with the results in this study. The effects of N rate on glucose concentrations in the stem and bud end of Dakota Russet have not been previously reported.

### 3.2. Concentrations of Acrylamide and Acrylamide Precursors

The cultivar by year interaction was significant for whole-tuber glucose concentration at 16 weeks of storage (Table 2). Due to high stem-end and low bud-end glucose concentrations in potato tuber, the whole-tuber glucose response to cultivar and year interaction was the same as that of stem-end glucose in Figure 1 (data not shown).

**Figure 4:**
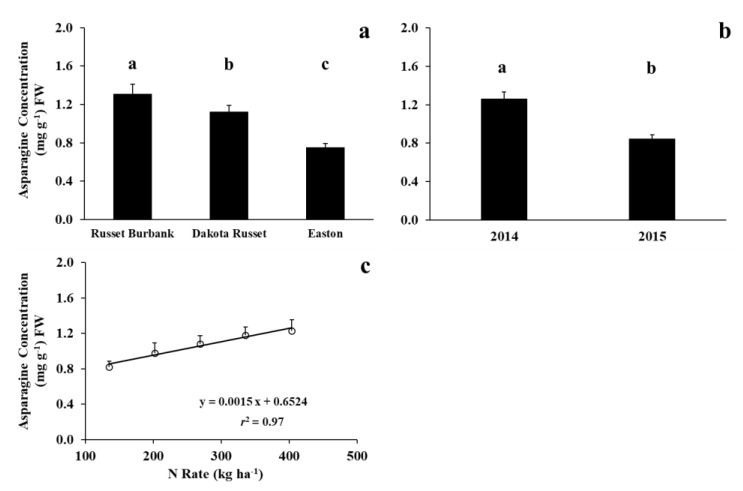
Main effects of cultivar (**a**), year (**b**), and N rate (**c**) on whole-tuber asparagine concentrations at 16-week storage (Means were separated at the 5% level, with the same letter above the bar indicating no significant difference.).

The main effects of cultivar, year, and N rate significantly affected whole-tuber asparagine concentrations (Table 2, Figure 4). Asparagine concentrations in Dakota Russet and Easton were consistently lower than those in Russet Burbank. Asparagine concentration increased with increasing N supply over three cultivars in two years. Averaged over cultivar and N rate, whole-tuber asparagine concentrations were lower in 2015 than in 2014, indicating the pronounced growing-condition effect. Warm weather early and late in the growing season in 2015 was favorable for tuber bulking and N uptake, but not for asparagine accumulation, suggesting that high tuber N did not proportionally convert to tuber asparagine.

**Figure 5:**
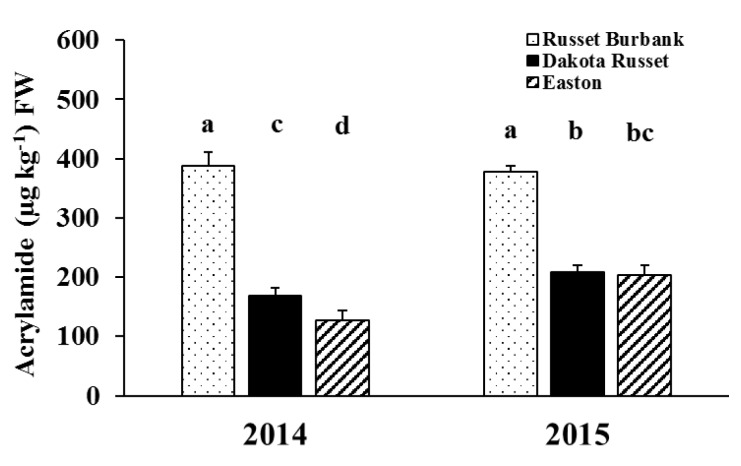
Cultivar by year interaction effect on acrylamide concentration at 16-week storage (Means were separated at the 5% level, with the same letter above the bar indicating no significant difference.).

The effect of cultivar by year interaction was significant on acrylamide concentration after 16 weeks of storage (Table 2 and Figure 5). Russet Burbank had the same level of acrylamide concentrations in two years (388 µg kg^−1^ in 2014 and 378 µg kg^−1^ in 2015). New cultivars Dakota Russet and Easton had significantly lower acrylamide concentrations than Russet Burbank in both years. However, the acrylamide concentration was significantly higher in Dakota Russet (169 µg kg^−1^) than in Easton (127 µg kg^−1^) in 2014, but on an equivalent level in 2015 (209 and 203 µg kg^−1^ for Dakota Russet and Easton, respectively). The lower acrylamide concentrations in French fries produced from Dakota Russet and Easton relative to Russet Burbank are consistent with reports from the North Dakota and Maine potato breeding programs [45,46].

**Figure 6:**
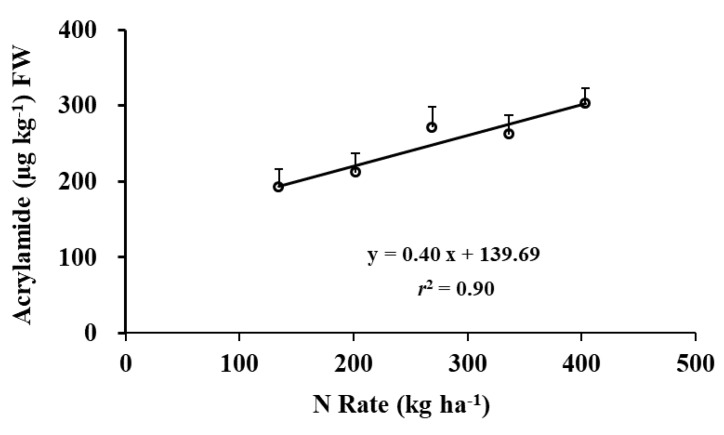
N rate effect on acrylamide concentration after 16 weeks of storage.

Averaged over cultivar and year, acrylamide concentrations increased linearly with increasing N rate (Figure 6), which is consistent with the conclusion from previous studies on Russet Burbank by Muttucumaru et al. [10] and Easton by Gause [44]. However, in another study, acrylamide concentrations in Russet Burbank were not affected by N supply in one year, and quadratically changed in the following year [25]. These results suggest acrylamide response to N rate probably depends on environmental conditions during the growing season and cultivar. For example, acrylamide concentrations decreased in Saturna and Hermes, and increased in Lady Rosetta and Markies with increasing N supply in the United Kingdom [10] but were not affected by N supply in Switzerland for the same four cultivars [5]. The effect of N rate on acrylamide concentrations for Dakota Russet has not been previously reported.

### 3.3. Relationships between Acrylamide, Glucose, and Asparagine

**Figure 7:**
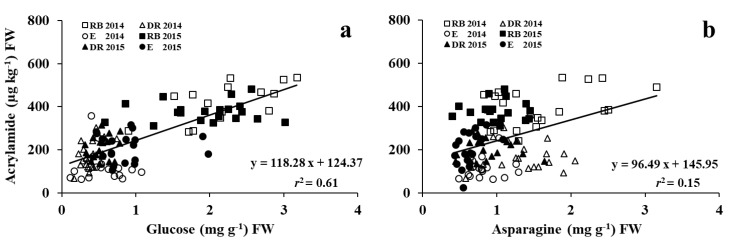
Relationships between acrylamide and glucose (**a**) or asparagine (**b**) concentrations of Russet Burbank, Dakota Russet, and Easton after 16 weeks of storage in 2014 and 2015.

The tuber glucose concentration was significantly correlated with the concentration of acrylamide in French fries over three cultivars in two years with *r*^2^ = 0.61 (Figure 7a).

The relationship between tuber asparagine and acrylamide was also significant, but not as strong (*r*^2^ = 0.15, *p* < 0.01, Figure 7b). Previous studies have reported similar conclusions of a strong correlation between reducing sugars and acrylamide with R^2^ values ranging from 0.73 to 0.95 [15,23,27,32,36], and a weak relationship between asparagine and acrylamide with R^2^ values ranging from 0.03 to 0.27 [23,27,36].

**Figure 8:**
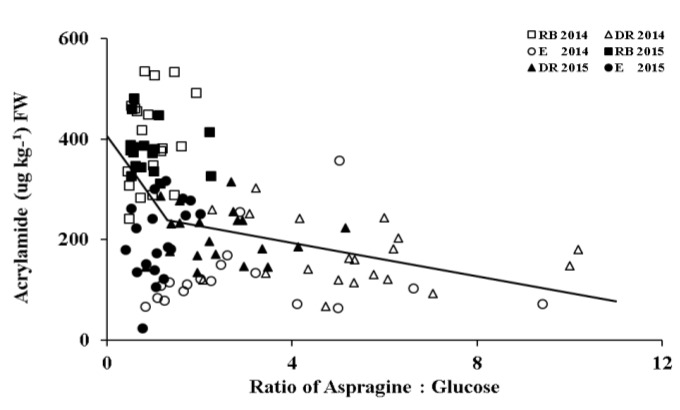
Relationship between acrylamide and the molar ratio of asparagine to glucose. Equations: y = 407.30 − 129.00x, x ≤ 1.306; y = 260.46 − 16.60x, x > 1.306.

While reducing sugar was considered the limiting factor in acrylamide formation, asparagine may play a more important role under certain circumstances [35,36]. Therefore, the relationship between acrylamide and the ratio of asparagine to glucose was analyzed using a piecewise linear regression (Figure 8). The equations for two linear lines are as follows:

acrylamide = 407.30 − 129.00 × asparagine/glucose for asparagine/glucose ≤ 1.306;(3)

acrylamide = 260.46 − 16.60 × asparagine/glucose for asparagine/glucose > 1.306.(4)

**Figure 9:**
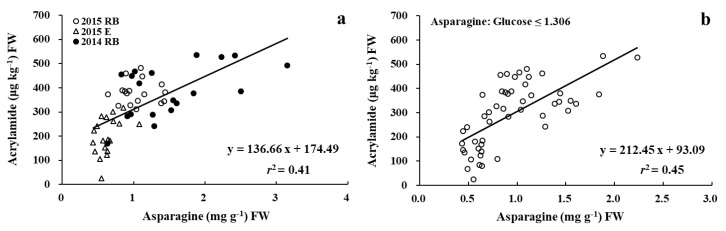
Relationships between acrylamide and asparagine (**a**) for Russet Burbank in two years and Easton in 2015, and (**b**) when asparagine/glucose ≤ 1.306.

When the asparagine/glucose ratio was less than 1.306, the correlation between acrylamide and asparagine was stronger, with *r*^2^ increased from 0.15 for all data (Figure 7) to 0.45 (Figure 9). The Asparagine correlation with acrylamide was much stronger with high reducing sugar accumulation. Previous studies have also reported that the relative concentrations of reducing sugar and asparagine could affect the correlation between asparagine, reducing sugar, and acrylamide: Matsuura-Endo et al. [30] observed a stronger correlation between asparagine and acrylamide (*r*^2^ = 0.68) when the molar ratio of fructose/asparagine was greater than 2. Muttucumaru et al. [24] reported a stronger correlation between asparagine and acrylamide, when concentrations of reducing sugars were 2.257-fold higher than asparagine concentrations.

In Figure 9a, most values of asparagine/glucose for Russet Burbank in two years and Easton in 2015 fell into the range of less than 1.306 as shown in Figure 9b, demonstrating a strong correlation between acrylamide and asparagine (*r*^2^ = 0.41). The ratio of asparagine/glucose was greater than 1.306 for Dakota Russet in both years and for Easton in 2014 (data not shown). No effects of N rate on asparagine/glucose ratio were observed at 16 weeks of storage. This result suggests that cultivar and environmental conditions during the growing season were more important than N rate in affecting the relative concentrations of reducing sugars and asparagine, and consequently their relationships with acrylamide.

### 3.4. Relationships between Fry Color, Glucose, and Acrylamide at 16 Weeks of Storage

**Figure 10:**
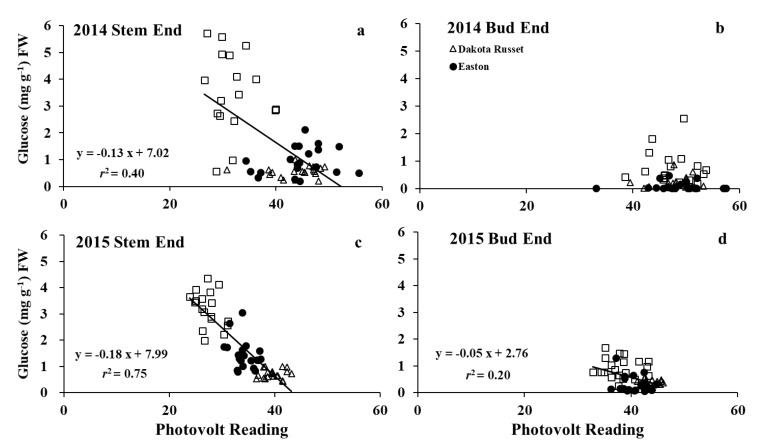
Relationships between tuber glucose and fry color at stem (**a**,**c**) and bud end (**b**,**d**) of French fries from tubers stored for 16 weeks at 7.8 °C in 2014 and 2015.

In the stem end, glucose concentration was significantly correlated with fry color with *r*^2^ = 0.40 and 0.75 in 2014 and 2015, respectively (Figure 10). However, it is not the case for the bud end. The correlation between bud-end glucose and fry color was not significant in 2014 and was weak in 2015 (*r*^2^ = 0.20). High stem-end sugar concentration generally caused dark color French fries (Figure 10a,c). Tubers containing the same amount of stem-end glucose had lower photovolt readings in 2015 than in 2014. Thus, although this correlation was significant for the stem end in both years, the equation in one year cannot be used to predict fry color or glucose concentration in another year. This is consistent with a previous study conducted with fry and chip cultivars [25].

**Figure 11:**
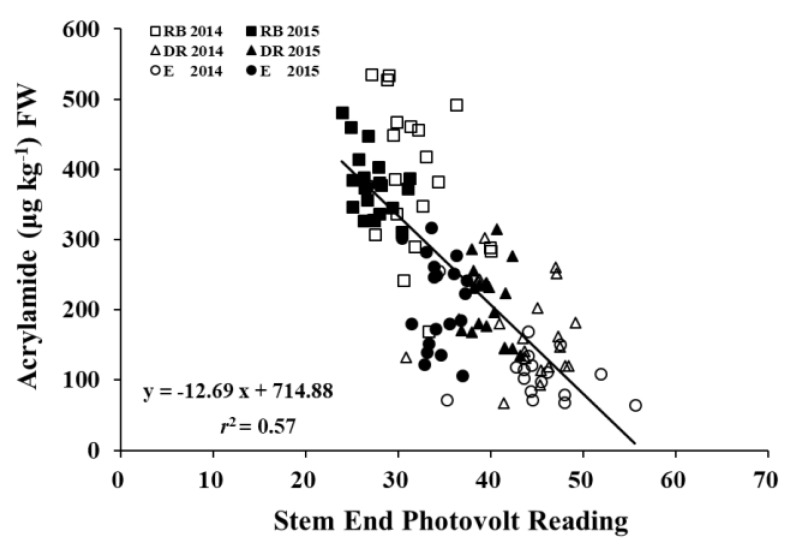
Relationship between stem-end fry color and acrylamide concentration of French fries from tubers stored for 16 weeks at 7.8 °C in 2014 and 2015.

The relationship between acrylamide and fry color was also investigated in this study. Bud-end glucose concentration was neglected due to the minimal contribution of bud-end glucose to fry color. Acrylamide and stem-end fry color was significantly correlated with an *r*^2^ = 0.57 and *p* < 0.05 over three cultivars and two years (Figure 11), indicating that fry color can be used as a predictor of acrylamide in fried potatoes. Fry color is considered acceptable when more than 80% of French fries have photovolt readings greater than 23 [47,48]. Therefore, all tested cultivars produced French fries with acceptable color in this study.

## 4. Conclusions

New cultivars Dakota Russet and Easton contained significantly lower concentrations of acrylamide precursors (glucose at both ends and asparagine), and consequently acrylamide concentrations as well. These results suggest that cultivar selection may be the most important consideration for minimizing acrylamide during potato processing. Environmental conditions during the growing season appeared to affect tuber glucose and asparagine concentrations in storage for all three cultivars. For example, glucose concentrations decreased during storage at both ends for all three cultivars in 2014 but increased or stayed the same depending on cultivar in 2015. Lower asparagine concentration in 2015 than in 2014 was observed in all three cultivars. Differences may have been due to colder conditions during harvest in 2014 than in 2015; although other stresses during the 2014 growing season cannot be ruled out.

While glucose is generally the limiting factor in acrylamide formation, asparagine could also play an important role under high reducing sugar accumulation. With asparagine/glucose ratio <1.306, asparagine was significantly correlated with the acrylamide concentration with *r*^2^ = 0.45. Cultivar and environmental conditions during the growing season seemed to affect the ratio of asparagine/glucose at 16 weeks of storage, while N rate showed no impact. Russet Burbank, Dakota Russet, and Easton produced French fries with acceptable fry color under the conditions of this study. Fry color can be used as a straightforward indicator of acrylamide and glucose concentrations in the stem end for test cultivars in this study, but the exact relationship between fry color and stem-end glucose concentration was found to differ by year.

## Figures and Tables

**Table 1:** The analysis of variance for glucose concentration during 32 weeks of storage in 2014 and 2015.

Source of Variance	Glucose During 32 Weeks of Storage	
	Stem End	Bud End
Main Effect		
Cultivar (C)	<0.0001	<0.0001
N Rate (N)	0.0718	0.4334
Year (Y)	0.1461	0.0018
Storage Time (S)	<0.0001	0.0765
Interactions		
N ×Y	0.3955	0.0142
N × S	0.3319	0.7266
S × Y	<0.0001	0.0052
C × Y	<0.0001	0.0049
C × N	0.0033	0.8609
C × S	<0.0001	0.2078
N × S × Y	0.5359	0.2544
C × N × Y	0.4159	0.1309
C × N × S	0.0562	0.6043
C × S × Y	0.0295	0.2783
C × N × S × Y	0.4247	0.6849

**Table 2:** The analysis of variance for the concentrations of glucose, asparagine, and acrylamide in 2014 and 2015.

Source	Whole-Tuber		
	Glucose (16 Weeks)	Asparagine (After Vine Kill)	Acrylamide (16 Weeks)
Main Effect			
Cultivar (C)	<0.0001	<0.0001	<0.0001
N Rate (N)	0.3042	0.0002	<0.0001
Year (Y)	0.3065	<0.0001	0.0052
Interactions			
C × N	0.1617	0.7709	0.4667
C × Y	<0.0001	0.3661	0.0049
N × Y	0.4420	0.4715	0.8615
C × N × Y	0.7170	0.9011	0.4493
